# Results of Serosurveillance and Forecasting the Third Wave of COVID-19 in an Industrial District in India

**DOI:** 10.7759/cureus.18097

**Published:** 2021-09-19

**Authors:** Deb Sanjay Nag, Minakshi Mishra, Rajan Chaudhry, Farah Rana, Sudhir Rai, Neelam Mehta, Minakshi Gupta

**Affiliations:** 1 Anaesthesiology, Tata Main Hospital, Jamshedpur, IND; 2 Pathology, Tata Main Hospital, Jamshedpur, IND; 3 Surgery, Tata Main Hospital, Jamshedpur, IND; 4 Biochemistry, Tata Main Hospital, Jamshedpur, IND; 5 Microbiology, Tata Main Hospital, Jamshedpur, IND

**Keywords:** prevalence study, covid-19 vaccination, covid-19 antibody positivity rate, covid-19, sars-cov-2

## Abstract

Prevalence of immunoglobulin G (IgG) severe acute respiratory syndrome coronavirus 2 (SARS-CoV-2) antibodies in the industrial district of East Singhbhum (Jharkhand, India) from July, August, November, and December 2020 and January 2021 after the first wave and in July 2021 after the second wave of coronavirus disease 2021 (COVID-19) infections may be utilized to find the possibility of a third wave of COVID-19 infections. Based on the trend of the loss of protective IgG antibodies after the first wave and the seropositivity of 75% in the district in July 2021, simple forecasting and proportional estimates of the seropositivity in the next eight months and the estimated maximum number of the cases was done. We also considered the seropositivity without vaccination in July 2021 (63%). Additionally, the trend of the weekly RT-PCR and rapid antigen testing for SARS-CoV-2 may also preemptively predict an imminent wave.

Based on the East Singhbhum population and the vaccination coverage with at least one dose till July 2021 (Covishield or Covaxin), it is estimated that a 4-5% monthly vaccination coverage rate of new individuals will not allow the seropositivity to fall below 50% and hold at bay a major wave. Vaccination coverage of 3% or less would allow a continuous drop in acquired immunity in the district and can potentially cause a rise in cases, making the community susceptible to a future surge of infections. A 3-5% vaccination rate of new individuals is unlikely to see a drop in the community seropositivity below 50% and the number of new cases of COVID-19 infections going above 478 to 712 per month at least till March 2022. The assumptions are based on presuming that there will be no new mutant of SARS-CoV-2 that escapes the immunity provided by previous infection or vaccination over the next eight months. However, currently, there is no evidence to speculate on any new variant of concern causing a major wave globally. The B.1.617.2 (delta) variant was first identified in October 2020 and there was a lag of six months to the second surge of COVID-19 infections in East Singhbhum, primarily caused by this variant. Additionally, 3% and above, with a rising weekly trend of reverse transcription-polymerase chain reaction (RT-PCR) positivity for SARS-CoV-2 can provide at least four to eight weeks advance warning before the peak of the wave if an imminent future wave is impending.

## Introduction

Various models like the susceptible-infected-recovered (SIR) model [1], the susceptible-exposed-infectious-recovered (SEIR) model [2], or the susceptible, undetected, tested (positive), and removed approach (SUTRA) model [3] are available in epidemiology to predict the outbreak of infectious diseases and have been used to estimate the future trend of the severe acute respiratory syndrome coronavirus 2 (SARS-CoV-2). However, due to the uncertainty of the disease spread, multiple unconsidered variables, including coronavirus disease (COVID)-appropriate behavior in the community and the evolving nature of the virus makes any long-term prediction difficult [1-3].

Before the availability of vaccines, the acquired immunity developed through clinical or subclinical infection with SARS-CoV-2 resulted in protective SARS‐CoV‐2 immunoglobulin G (IgG) antibodies. However, these antibodies decay over a period of weeks to months, again making the individual susceptible to infection [4]. It is speculated that when the IgG seropositivity falls below a certain threshold, the community is potentially again susceptible to another wave of the pandemic. Although SARS-CoV-2-specific IgG memory B cells and SARS-CoV-2-specific memory lymphocytes also provide antiviral immunity, their durability and effectiveness need further studies to assess their correlation to the protection [5]. Since the onset of SARS-CoV-2 vaccination, a longer-lasting acquired immunity is provided by all the above mechanisms. Currently, the susceptibility of a community to a surge of infection, thus overwhelming the healthcare infrastructure would depend on the acquired immunity developed either through previous infection or vaccination.

Based on the IgG seropositivity acquired through clinical or subclinical infection and its decline beyond two months, projections can be made about the next surge in COVID-19 infections in the community. However, if the vaccination coverage can be ramped up to neutralize the decrease in IgG seropositivity acquired through clinical or subclinical infection over a period to a cumulative acquired immunity above 70-75%, the community can prevent a large surge in infections.

This study presents the detailed findings of the prevalence of SARS-CoV-2 antibodies in July 2021 in an industrial district of East Singhbhum in the state of Jharkhand, India. We also correlate the findings of the previous prevalence of SARS-CoV-2 antibodies in the months of July, August, November, and December 2020 and January 2021 in the district along with the monthly incidence of new COVID-19 cases in the district to correlate with the vaccination coverage to suggest possible trajectories of COVID-19 infections in the future.

## Materials and methods

Institutional approval was taken to study the prevalence of IgG SARS-CoV-2 antibodies in the community in the industrial district of East Singhbhum in the state of Jharkhand, India, in July, August, November, and December 2020 and January 2021 for the first wave of COVID-19 infections and in July 2021 after the second wave of COVID-19 infections. Based on the trend of the available data, projections were made for the months whose data was not available based on the drop in IgG SARS-CoV-2 antibodies in the available data, its correlation with the incidence of infection in the district [6], and forecasting of the seroprevalence of IgG SARS-CoV-2 antibodies till March 2022. The data for the month-wise incidence of new cases [6] and immunization coverage [7] was utilized for making the projections. The seroprevalence of IgG SARS-CoV-2 antibodies in the unimmunized in July 2021 was utilized to understand the prevalence of IgG SARS-CoV-2 antibodies from clinical or sub-clinical acquired infection.

Three milliliters of a random venous sample were collected for SARS-CoV-2 IgG antibodies, maintaining the cold chain with a temperature of 15-20 degrees Celsius before being processed and tested. Samples were collected from the workplace, community, hospital, and clinics. The samples were allowed to clot and then centrifuged at 4500 rpm for five minutes to collect the serum separately. While the SARS-CoV-2 IgG assay with the Access SARS-CoV-2 IgG assay (Beckman Coulter, Brea, California) was done in July, August, November, and December 2020 and January 2021, it was done with the Access SARS-CoV-2 IgG II in July 2021. The Access SARS-CoV-2 IgG and Access SARS-CoV-2 IgG II assay (Beckman Coulter, Brea, California) detects antibodies to the receptor-binding domain (RBD) of the spike protein. Based on the manufacturer guidelines, automated chemiluminescent immunoassay (CLIA) with the Access SARS-CoV-2 IgG assays, the signal-to-cut-off (S/CO) ratio above 1.0 was considered positive, S/CO ratio between 0.8 and 1.0 was considered equivocal, and S/CO ratio below 0.8 was considered negative for SARS‐CoV‐2 IgG antibodies. The Access SARS-CoV-2 IgG assay is reported to have 100% sensitivity and 99.8% specificity [4]. Similarly, with the Access SARS-CoV-2 IgG II assay, measured light units are converted to arbitrary units using the calibration curve; ≥ 10 AU/mL was considered positive, and < 10 AU/mL was considered negative for SARS‐CoV‐2 IgG antibodies. Access SARS-CoV-2 IgG II assay is reported to have 100% sensitivity and specificity [8]. The protective effect of the COVID-19 vaccine in India is derived from the existing literature, which suggests that even one dose is protective up to 61% from developing an infection, 70% from hospitalization, 96% from the need for oxygen therapy, and 95% from the need for intensive care unit admission [9].

It was hypothesized based on available literature that the SARS-CoV-2 IgG antibodies acquired by clinical or sub-clinical infections disappear over a period of time, however, COVID-19 vaccination provides a longer-lasting immunity [10]. If the community vaccination as able to ramp up and ensure coverage to cover the drop in SARS-CoV-2 IgG antibodies acquired by clinical or sub-clinical infection, the community immunity can be ramped up for coverage above a certain threshold to ensure no major wave or surge of COVID-19 unless there is a major mutation resulting in immune escape from immunity acquired by previous infection or vaccination.

Genomic sequencing of 57 SARS-CoV-2 positive samples by reverse transcription-polymerase chain reaction (RT-PCR) positive by the STANDARD M nCoV Real-Time Detection kit (SD Biosensor, Republic of Korea) in April 2021 and 40 samples in June 2021 from the Tata Main Hospital, Jamshedpur (in East Singhbhum district) were done at the Institute of Life Science (ILS), Bhubaneswar (India), to understand the SARS-CoV-2 lineage responsible for most of the infections in the second wave of the COVID-19 infection.

The positivity of the RT-PCR samples for SARS-CoV-2 amongst a mix of diseased and asymptomatic close contacts of COVID-19 positive and those with a travel history outside the district/state was monitored. Additionally, the positivity of rapid, point‐of‐care antigen-based tests (RAT) for diagnosis of SARS‐CoV‐2 infection amongst asymptomatics was also monitored for the trend. Both these trends allow us to estimate how early before the onset of a peak can the surge in cases be predicted. 

Statistical analysis

Data were maintained in Microsoft Excel (Microsoft® Corp., Redmond, WA), analyzed through the forecasting tools available in it, and a proportional estimation was performed based on previous months' data.

## Results

Table 1 shows the number of new COVID-19 cases detected in the district of East Singhbhum (Jharkhand, India) and the IgG SARS-CoV-2 seropositivity in the community (monthly random sample size 1000-4575 individuals). While seropositivity for July, August, November, and December 2020 and January 2021 for the first wave of COVID-19 infections and in July 2021 after the second wave of COVID-19 infections were based on actual serosurveillance data; the projections for the remaining months were derived based on proportionate decrease based on previous months and forecasting on Microsoft Excel. The district has an estimated population of 27,88,488 [11] and based on the data available through the CoWIN portal on July 31, 2021, cumulatively, 25% of the population had received at least one dose of the COVID-19 vaccine [7].

**Table 1:** Monthly incidence of COVID-19, IgG SARS-CoV-2 antibody positivity, and percentage of the population receiving at least one dose of COVID-19 vaccine in East Singhbhum district

Month	Monthly incidence of COVID-19 in East Singhbhum	IgG SARS-CoV-2 antibody positivity	Determinants for IgG SARS-CoV-2 antibody positivity	Percentage of the population covered by at least one dose of COVID-19 vaccine
May-20	106	-	-	-
Jun-20	286	-	-	-
Jul-20	1525	7%	Actual serosurveillance data	-
Aug-20	5161	11%	Actual serosurveillance data	-
Sep-20	7188	20%	Proportionate decrease based on previous months	-
Oct-20	1998	25%	Proportionate decrease based on previous months	-
Nov-20	937	30%	Actual serosurveillance data	-
Dec-20	715	24%	Actual serosurveillance data	-
Jan-21	399	20%	Actual serosurveillance data	0.07%
Feb-21	220	15%	Forecasting based on Microsoft Excel	0.14%
Mar-21	660	14%	Forecasting based on Microsoft Excel	3.11%
Apr-21	14694	19%	Forecasting based on Microsoft Excel	8.26%
May-21	15639	39%	Forecasting based on Microsoft Excel	18.75%
Jun-21	2010	72%	Proportionate decrease based on previous months	21.45%
Jul-21	217	75%	Actual serosurveillance data	25.21%

**Figure 1:**
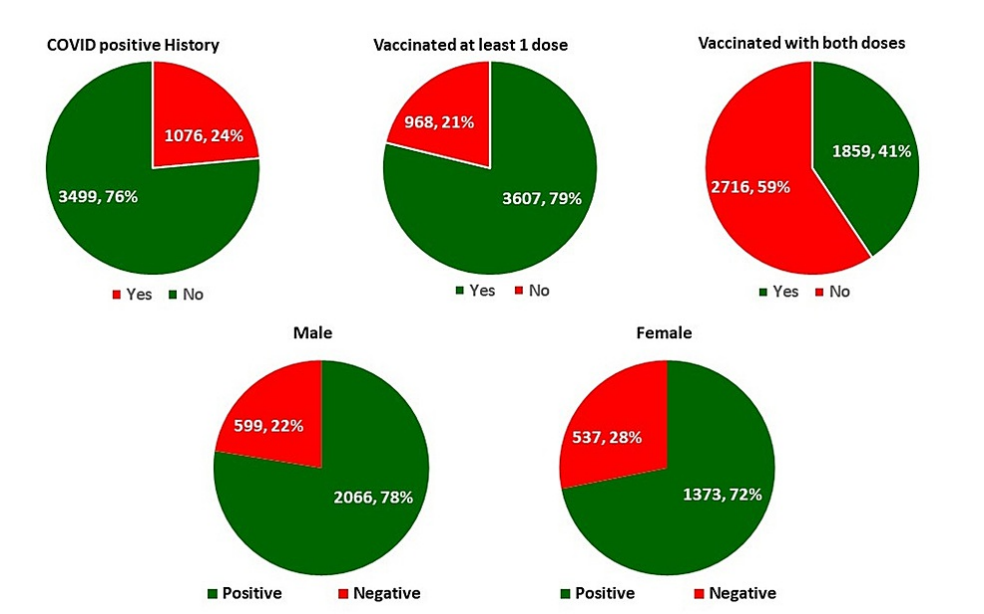
Findings of IgG SARS-CoV-2 serosurveillance in East Singhbhum (Jharkhand, India)

After the surge in cases (April-May 2021) in the district (30,333 cases in a population of approximately 27,88,488 in the district) [6-7], the IgG SARS-CoV-2 antibody positivity was 75% in July 2021. While 24% of the surveyed population in July 2021 had a laboratory-proven COVID-19 history, 79% of the surveyed population had received at least one dose, and 41% of the surveyed population had received both doses of a COVID-19 vaccine. The IgG SARS-CoV-2 seropositivity was 6% higher in males as compared to females (78% vs 72%). The seropositivity amongst the non-vaccinated (n=968) was significantly lower (63%) as compared to those who have received at least one dose (n=3607) (78%). The findings are represented in Figure 1. The seropositivity amongst the unvaccinated represents the seroconversion due to clinical or sub-clinical infection. Similarly, a 14% laboratory-proven history of previous COVID-19 infections amongst the 1,136 IgG SARS-CoV-2 negative individuals represents a loss of protective antibodies as evidenced in previous studies.

**Figure 2:**
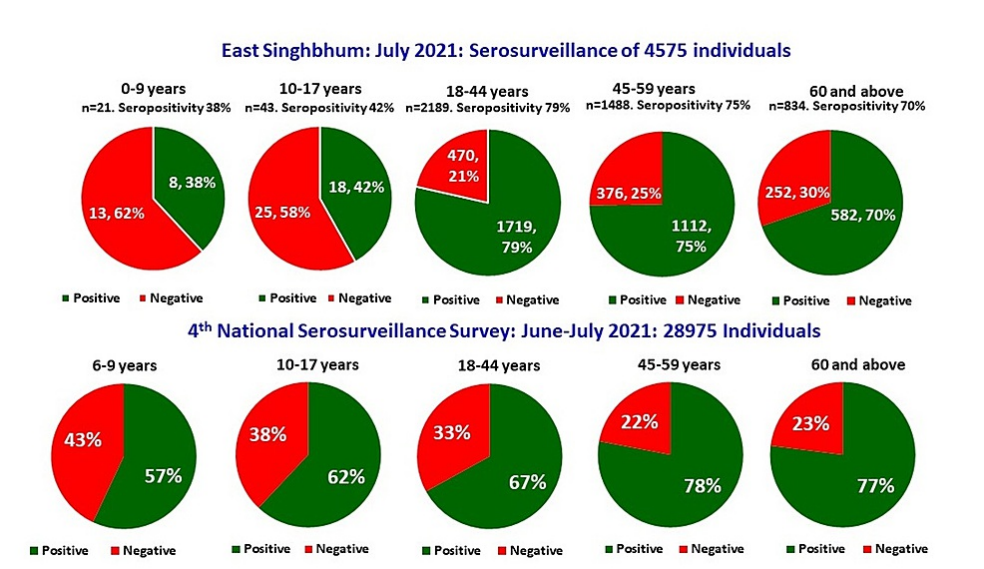
Seroprevalence of IgG SARS-CoV-2 seropositivity based on age (years) in East Singhbhum as compared with the nation (4th National Serosurveillance, Indian Council of Medical Research [ICMR], June-July 2021)

The findings of the serosurveillance were compared to the 4th National Serosurveillance conducted by the Indian Council of Medical Research (ICMR) in June-July 2021, across India. The seroprevalence of the IgG SARS-CoV-2 seropositivity was found out to be higher in East Singhbhum (75%) as compared to the country (68%) [12]. The age comparisons of both surveys are shown in Figure 2.

Genomic sequencing of 57 SARS-CoV-2 RT-PCR positive samples by RT-PCR (April 2021) and 40 samples (June 2021) from the Tata Main Hospital, Jamshedpur (in East Singhbhum), were done at the Institute of Life Science (ILS), Bhubaneswar (India). Of the samples from April 2021, which was at the peak of the second wave of the pandemic in the district, 75% of the samples were found to be of the B.1.617.2 variant and 90% of the samples from June 2021 were found to be of the B.1.617.2 variant. This establishes the fact that most of the COVID-19 cases in East Singhbhum during the second wave were of the B.1.617.2 (WHO label Delta) variant.

Based on the hypothesis that the IgG SARS-CoV-2 positivity was acquired either through infection (shorter lasting) or through vaccination (longer lasting) [10], predictions were derived for future immunity based on dropping seropositivity acquired by infection and ramp-up of vaccination in the district. Estimates were taken for 3%, 4%, and 5% coverage of the district with at least one dose of the COVID-19 vaccine. Based on these assumptions, projections were derived for the possible number of COVID-19 cases in East Singhbhum till March 2022 as depicted in Table 2. The immunity in the community is defined by the formula: Percentage immunity in the district = Immunity developed through acquired infection among unimmunized plus Immunity developed through vaccination.

**Table 2:** Actual and estimated seropositivity due to COVID-19 infection only, percentage immunity with variable vaccination coverage, and maximum number of cases possible with that vaccination coverage

Month	Seropositivity due to infection only	Percentage immunity in the district if at least 5% of new individuals receive vaccinations every month	Percentage Immunity in the community if at least 4% receive vaccinations every month	Percentage Immunity in the community if at least 3% receive vaccinations every month	Maximum number of infections possible in the district if at least 5% of new individuals receive vaccinations every month	Maximum number of infections possible in the district if at least 4% of new individuals receive vaccinations every month	Maximum number of infections possible in the district if at least 3% of new individuals receive vaccinations every month
May-20					106	106	106
Jun-20					286	286	286
Jul-20	7.40%	7.40%	7.40%	7.40%	1525	1525	1525
Aug-20	10.60%	10.60%	10.60%	10.60%	5161	5161	5161
Sep-20	20.15%	20.15%	20.15%	20.15%	7188	7188	7188
Oct-20	24.93%	24.93%	24.93%	24.93%	1998	1998	1998
Nov-20	29.71%	29.71%	29.71%	29.71%	937	937	937
Dec-20	24.47%	24.47%	24.47%	24.47%	715	715	715
Jan-21	20.33%	20.33%	20.33%	20.33%	399	399	399
Feb-21	15.36%	15.36%	15.36%	15.36%	220	220	220
Mar-21	14.41%	15.82%	15.82%	15.82%	660	660	660
Apr-21	18.84%	24.28%	24.28%	24.28%	14694	14694	14694
May-21	35.68%	54.46%	54.46%	54.46%	15639	15639	15639
Jun-21	64.04%	62.94%	62.94%	62.94%	2010	2010	2010
Jul-21	63.00%	75.00%	75.00%	75.00%	217	217	217
Aug-21	51.90%	66.43%	65.95%	65.47%	478	485	491
Sep-21	43.11%	63.14%	62.00%	60.86%	524	541	557
Oct-21	32.57%	59.69%	57.66%	55.64%	574	602	631
Nov-21	30.57%	61.96%	59.18%	56.40%	541	581	620
Dec-21	21.04%	60.69%	56.74%	52.79%	559	616	672
Jan-22	14.69%	61.79%	56.67%	51.55%	544	617	689
Feb-22	8.84%	63.73%	57.35%	50.97%	516	607	698
Mar-22	1.50%	65.74%	57.86%	49.98%	488	600	712

The percentage immunity and the maximum number of new infections in the district are the actual values up to July 2021 (and is agnostic of the fact that whether new 3%, 4%, or 5% of the community receives at least one dose of COVID-19 vaccine in future months). The values from August 2021 onwards are based on forecasting and proportionate projections based on a drop in IgG seropositivity amongst the unimmunized and a ramp-up of immunization of the district. While the drop in seropositivity is based on the natural drop observed in actual serosurveillance surveys in the district in November 2020, December 2020, and January 2021 and available medical literature [4], the incidence of new cases is dependent on the available evidence of COVID-19 infection in subjects who have received at least one dose of COVID-19 vaccine in India [9]. While a 5% new individual vaccination coverage in the district every month would predict a minimum immunity of 59.69% in the community in October 2021 and a 4% new individual vaccination coverage in the district every month would predict a minimum immunity of 56.67% in the community in January 2022, a 3% new individual vaccination coverage would ensure a continuous drop in minimum immunity community to below 50% in March 2022. Based on these predictions, a 3-5% vaccination coverage of new individuals is unlikely to see a drop in the community seropositivity below 50% and the number of new cases going above 478 to 712 per month. The estimates are based on the fact that in July 2021 in East Singhbhum, the seropositivity for IgG SARS-CoV-2 was 75% in the community and 63% in those who have not received even one dose of the COVID-19 vaccine. This led to the hypothesis that 63% of the community in the East Singhbhum district developed IgG SARS-CoV-2 due to previous infection and cumulative seropositivity of 75% was achieved based on previous infection and vaccination. While a monthly 4-5% vaccination coverage of the district beyond July 2021 would ensure that the seropositivity never falls below 57%, only with vaccination coverage of 3% or less, there would be a risk of rising cases and a future wave.

**Figure 3:**
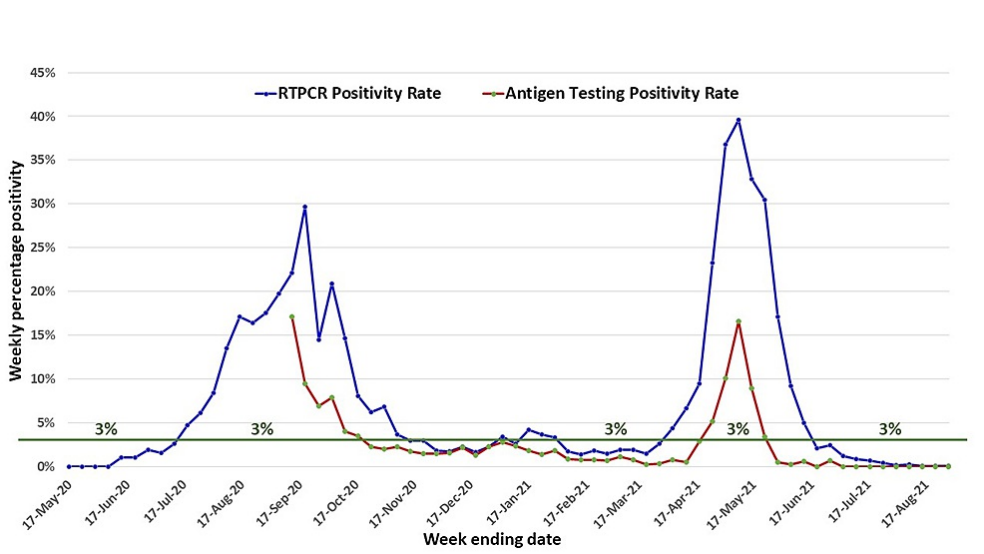
Weekly RT-PCR and RAT positivity at the Tata Main Hospital, Jamshedpur (in East Singhbhum district) RT-PCR: reverse transcription-polymerase chain reaction; RAT: rapid, point‐of‐care antigen-based test

However, the incidence of SARS-CoV-2 variants as observed during the first and second waves of COVID-19 in East Singhbhum, a rising weekly positivity of the RT-PCR samples for SARS-CoV-2 and rapid, point‐of‐care antigen-based tests (RAT) for the diagnosis of SARS‐CoV‐2 infection can pre-emptively predict a surge in infections. Figure 3 depicts the weekly RT-PCR and RAT positivity of the samples tested by Tata Main Hospital (TMH), Jamshedpur, in the East Singhbhum district. Till August 29, 2021, TMH has done 1,35,545 RT-PCR & 1,17,772 RAT tests, indicating the adequacy of the sample size. This clearly shows that based on the virulence of the SARS-CoV-2 virus strain, a rising trend of RT-PCR positivity above 3% weekly gives a six-week (wave 2 caused predominantly by the B.1.617.2 (WHO label Delta) variant) to 10-week (in wave one) window before it hits the peak of the epidemic. The rising RT-PCR positivity is a better lead indicator of an impending wave as compared to RAT.

## Discussion

There is substantial literature evidence of rapid loss of protective IgG antibodies, starting as early as two months after acute infection [13]. Naturally acquired antibodies decrease at different rates after infection with SARS-CoV-2 [4,14-15]. Observing its trend with the corresponding surge in infections can predict the future course in the community. While various predictive models exist, most of the mathematical models are derived based on the susceptible community, rate of rise and fall in infections, as well as the presumed immunity in the community. This is the first model based on actual serology data in a district, its trends, and vaccination coverage ramp-up. There is evidence available to show that that the immunity provided by COVID-19 vaccination is better and longer-lasting than that acquired after infection with SARS-CoV-2 [10]. Earlier reported reviews had concluded that there is a need for more robust seroprevalence data of IgG antibodies to predict the possible development of herd immunity [16]. It was suggested that “evidence-based and targeted public health measures informed by accurate real-world data” [16] would help us proactively plan to mitigate the challenges posed by the COVID-19 pandemic. Our analysis shows that in a district with 75% seropositivity of IgG antibodies, based on the existing population, it is possible to predict the percentage of the population that needs to be covered every month to prevent a surge in infections. The analysis shows, that in the East Singhbhum district (Jharkhand, India), with a seroprevalence of 75% IgG positivity, at least 4-5% of the population needs to be covered with at least one dose of vaccination (and subsequently the second dose) to prevent the seropositivity from dropping below 50%.

The delta (B.1.617.2) variant has now emerged and outcompeted the earlier rapidly spreading variants of concern (VOC) across the globe and is responsible for most infections globally and in India [17]. With whole-genome sequencing (WGS) of SARS-CoV-2 establishing that 75-90% of the COVID-19 infections in East Singhbhum district during the second wave were due to the B.1.617.2 or delta variant, it establishes the fact that a large majority of the community now has protective antibodies against the current globally dominant variant. The variant is considered to be 50% more transmissible than the alpha (B.1.1.7) variant [18], a higher secondary attack rate of 3% [19], and a modest reduction in vaccine effectiveness [19]. Based on this, it can reasonably be concluded that unless there is an emergence of a new variant of concern that escapes the immunity provided by previous infection (predominantly with the B.1.617.2 variant) or vaccination, there is unlikely to be a major surge of infection within the next six months if 4-5% of the East Singhbhum district continues to be vaccinated every month. While the predicted maximum number of infections range between 217-712 until March 2022, the actual numbers would depend upon COVID-appropriate behavior, travel restrictions, and extent of lockdown.

While there are many models to predict a surge in infections, a simplistic plotting of the week-on-week RT-PCR positivity for SARS-CoV-2 is better than RAT positivity and can pre-emptively predict the peak at least four to eight weeks ahead. Data from Tata Main Hospital, Jamshedpur (India), the largest tertiary care hospital in the studied district (East Singhbhum), show that a 3% positivity and a rising trend, can predict the onset up to four to eight weeks in advance based on the virulence of the virus. To date, there is very little literature evidence correlating the capability of RAT and RT-PCR positivity for SARS-CoV-2 to suggest the onset of a surge in COVID-19 infections.

One of the limitations of this analysis is that although similar in sensitivity and specificity, variability in the test kits used in the SARS-CoV-2 IgG assay during the various phases of the serosurveillance study can give variable results. While there is adequate evidence that immune protection against other human coronaviruses (HCoV) is not long-lasting, contrary evidence is also available showing the presence of potent “neutralizing activity even in the absence of detectable serum immunoglobulin G (IgG)” [15]. Additionally, besides the SARS-CoV-2 IgG antibodies, B cells and T cells also contribute to the immunity against COVID-19 infections [20]. However, the prediction model looks at the worst-case scenario to enable the community to ensure healthcare preparedness and long-lasting immunity may provide an even higher level of protection. The actual number of cases and hospitalization can be even lower. Another limitation of this analysis and prediction model is the fact that it has been presumed that there will be no new mutant of SARS-CoV-2 that escapes the immunity provided by previous infection or vaccination. The projected maximum number of cases from August 2021 to March 2022 is based on the limited evidence of the study on protective immunity provided by a single dose of the COVID vaccines available in India [9]. However, with increasing coverage and availability of many types of vaccines, we will better understand the immunity provided by the various vaccines. Additionally, the ramp-up of the second dose of the COVID vaccination could potentially result in further avoidance of an imminent third wave.

## Conclusions

Based on the trend of seropositivity of the IgG SARS-CoV-2 antibody in a defined population and the trend of vaccination coverage, it is possible to predict the future trend and possibility of a surge in COVID-19 infections. This will give the health authorities and community an understanding of the percentage of the community that needs to be covered by vaccination every month to prevent future waves, provided there is no other variant of concern that escapes the immunity provided by previous COVID-19 infection or vaccination. Whole-genome sequencing (WGS) of SARS-CoV-2 enables the health care providers within the community to understand the incidence of the predominant COVID-19 variant and understand the susceptibility of the community to the prevalent variants of concern in the future. A trend of RT-PCR positivity for SARS-CoV-2 can establish that if the positivity is 3% and rising, it can predict the peak of a future wave four to eight weeks in advance. However, based on the seropositivity due to previous infection and vaccination coverage, it is more likely that the COVID-19 pandemic has entered a phase of endemicity where a simmering low level of transmission would continue amongst the susceptible for the next few months and years. However, increasing vaccination coverage would ensure a reduced need for hospital beds and critical care facilities.
